# LuxR solos in *Photorhabdus* species

**DOI:** 10.3389/fcimb.2014.00166

**Published:** 2014-11-18

**Authors:** Sophie Brameyer, Darko Kresovic, Helge B. Bode, Ralf Heermann

**Affiliations:** ^1^Bereich Mikrobiologie, Biozentrum, Ludwig-Maximilians-Universität MünchenMünchen, Germany; ^2^Fachbereich Biowissenschaften, Merck Stiftungsprofessur für Molekulare Biotechnologie, Goethe-Universität FrankfurtFrankfurt am Main, Germany

**Keywords:** LuxR solos, quorum sensing, cell-cell communication, quorum quenching, entomopathogenic bacteria

## Abstract

Bacteria communicate via small diffusible molecules to mediate group-coordinated behavior, a process designated as quorum sensing. The basic molecular quorum sensing system of Gram-negative bacteria consists of a LuxI-type autoinducer synthase producing acyl-homoserine lactones (AHLs) as signaling molecules, and a LuxR-type receptor detecting the AHLs to control expression of specific genes. However, many proteobacteria possess one or more unpaired LuxR-type receptors that lack a cognate LuxI-like synthase, referred to as LuxR solos. The enteric and insect pathogenic bacteria of the genus *Photorhabdus* harbor an extraordinarily high number of LuxR solos, more than any other known bacteria, and all lack a LuxI-like synthase. Here, we focus on the presence and the different types of LuxR solos in the three known *Photorhabdus* species using bioinformatics analyses. Generally, the N-terminal signal-binding domain (SBD) of LuxR-type receptors sensing AHLs have a motif of six conserved amino acids that is important for binding and specificity of the signaling molecule. However, this motif is altered in the majority of the *Photorhabdus*-specific LuxR solos, suggesting the use of other signaling molecules than AHLs. Furthermore, all *Photorhabdus* species contain at least one LuxR solo with an intact AHL-binding motif, which might allow the ability to sense AHLs of other bacteria. Moreover, all three species have high AHL-degrading activity caused by the presence of different AHL-lactonases and AHL-acylases, revealing a high quorum quenching activity against other bacteria. However, the majority of the other LuxR solos in *Photorhabdus* have a N-terminal so-called PAS4-domain instead of an AHL-binding domain, containing different amino acid motifs than the AHL-sensors, which potentially allows the recognition of a highly variable range of signaling molecules that can be sensed apart from AHLs. These PAS4-LuxR solos are proposed to be involved in host sensing, and therefore in inter-kingdom signaling. Overall, *Photorhabdus* species are perfect model organisms to study bacterial communication via LuxR solos and their role for a symbiotic and pathogenic life style.

## Introduction

*Photorhabdus* spec. are pathogenic enteric bacteria that maintain a mutualistic interaction with heterorhabditid nematodes and can infect a wide variety of insect species. To date, three different *Photorhabdus* species are known: *P. luminescens*, *P. temperata*, and *P. asymbiotica* (Fischer-Le Saux et al., 1999). Whereas the first two species are highly pathogenic toward insects, *P. asymbiotica* additionally infects men (Gerrard et al., 2003). *P. asymbiotica* is associated with severe soft-tissue and systemic infections in humans, and is considered as an emerging threat (Gerrard et al., 2004). Commonly, all *Photorhabdus* bacteria colonize the gut of the infective juvenile stage of *Heterorhabditis* spec. nematodes. Upon entering insect larvae, the nematodes directly inject the bacteria by regurgitation into the insect's hemocoel. Once inside the insect, the bacteria rapidly replicate and quickly establish a lethal septicemia in the host by production of a broad range of different toxins that kill the insect within 48 h. At this stage the dying insect glows, due to bacterial luciferase production. Bioconversion of the insect's body by a huge set of bacterial exoenzymes produces a rich food source both for the bacteria and the nematodes. Furthermore, the bacteria support nematode reproduction probably by providing essential nutrients that are required for efficient nematode proliferation (Han and Ehlers, 2000). Moreover, the bacteria produce several antibiotics to defend the insect cadaver from invasion of other bacteria. When the insect cadaver is depleted, the nematodes and bacteria re-associate and emerge from the carcass in search of a new insect host (see Clarke, 2008; Waterfield et al., 2009, for review). During this complex life cycle the bacteria constantly have to monitor their host environment, and to communicate with each other as well as with their hosts to perfectly adapt to the respective conditions.

Bacterial communication via small molecules to mediate group-coordinated behavior, referred to as quorum sensing (QS), is well recognized. Typically, Gram-negative bacteria use small diffusible molecules, e.g., acyl-homoserine lactones (AHLs) derived from fatty acids, whereas Gram-positive bacteria use peptide derivatives for communication. However, recently the production of AHLs by a Gram-positive bacterium belonging to the *Exiguobacterium* genera isolated from marine water was discovered (Biswa and Doble, 2013). The prototypical quorum sensing system of Gram-negative bacteria consists of a LuxI-like autoinducer synthase that produces AHLs as signals and a LuxR-type receptor that detects the AHLs to control expression of specific genes (Waters and Bassler, 2005). The AHLs are constantly synthesized by LuxI, and are sensed by the cognate LuxR-like receptor when exceeding a threshold concentration. Upon AHL-binding, LuxR binds to the promoter/operator regions of the target genes/operons causing altered gene expression in response to the cell count. Thus, bacteria respond to AHLs and adapt bacterial group-behavior by regulation of gene expression when the cell density reaches a certain size (quorum). Signaling via AHLs has been linked to diverse phenotypes like the production of virulence factors, motility, antibiotic production, sporulation, bioluminescence or biofilm formation (Waters and Bassler, 2005). LuxI/LuxR based quorum sensing systems have been intensively studied. The first system was described in *V. fischeri* showing that AHLs are used to regulate light production dependent on the cell density (Nealson and Hastings, 1979). Furthermore, LuxR-based cell-cell communication is medically relevant as many pathogenic bacteria use these quorum sensing systems for an effective infection process (Rutherford and Bassler, 2012).

Moreover, many bacterial genomes encode additional LuxR homologs lacking a cognate LuxI synthase. These LuxR homologs are designated as LuxR orphans (Patankar and González, 2009) or LuxR solos (Subramoni and Venturi, 2009). Genome sequencing of 265 proteobacterial genomes revealed that 68 of these encode at least one LuxI and one LuxR homolog (Case et al., 2008). Furthermore, 45 of these 68 genomes encode more LuxR-type proteins than LuxI homologs like *Pseudomonas aeruginosa*, which harbors beside the two classical QS systems, LasI/LasR and RhlI/RhlR, the LuxR solo QscR (Oinuma and Greenberg, 2011). Further 45 genomes do not harbor a *luxI*-like AHL synthase encoding gene, but encode at least one LuxR homolog. The three *Photorhabdus* species all lack a LuxI homolog, but have an extraordinary high number of genes that encode potential LuxR solos and are therefore assumed to have a huge capacity for cell-cell and/or inter-kingdom communication (Heermann and Fuchs, 2008). Therefore, these bacteria are perfect model organisms to study bacterial cell-cell communication via LuxR solos. Here we focus on the function of the multiple LuxR solos of the three known *Photorhabdus* species for their life cycle, their role in cell-cell communication and inter-kingdom signaling, as well as the specificity of signal perception.

## Materials and methods

### Bacterial strains

The strains *Photorhabdus luminescens* TT01 (Fischer-Le Saux et al., 1999), *Photorhabdus temperata* NC19 (Tailliez et al., 2010), *Photorhabdus asymbiotica* ATCC43949 (Wilkonson et al., 2009), and *Vibrio harveyi* BB120 (Bassler et al., 1997) were used in this study.

### Quorum quenching bioassay

Bioluminescence of *Vibrio harveyi* wild-type (BB120) was used as read-out to analyze degradative activity of AHLs in *Photorhabdus* species supernatants. Therefore, *V. harveyi* BB120 strain was grown at 30°C in LM medium [10% (w/v) peptone, 5% (w/v) yeast extract, 20% (w/v) NaCl] supplemented with carbenicillin (100 μg/ml). An overnight culture of *V. harveyi* BB120 was inoculated at Optical Density (OD) at 600 nm = 0.2 in LM and a volume of 80 μl was pipetted into each well of a 96-microtiter plate. Cells were then grown for 4.5 h aerobically at 30°C until the mid-exponential growth phase, the growth phase where bioluminescence naturally occurs (Anetzberger et al., 2012). After 4.5 h of growth a volume of 80 μl of the cell-free supernatant of *P. luminescens*, *P. temperata*, or *P. asymbiotica*, respectively, was added. Thus, *P. luminescens* and *P. temperata* cultures were grown at 30°C, and *P. asymbiotica* culture was grown at 37°C in CASO Medium. After 4 d and 7 d, cells were adjusted to OD 600 nm = 1 before the supernatant was harvested via centrifugation. As control the same volume of LM medium and supernatant of a *V. harveyi* BB120 culture, harvested after 8 h and 1 day of growth, was added. OD and luminescence were monitored every hour with a Sunrise plate reader (Tecan, Crailsheim) and a Centro luminometer (Berthold Technologies, Bad Wildbad), respectively.

### Bioinformatics studies

For bioinformatics identification of LuxR solos the genome assembly and annotation reports of *Photorhabdus luminescens* subsp. *laumondii* TT01 (NCBI reference sequence NC_005126.1), of *Photorhabdus temperata* subsp. *khanii* NC19 (BioSample SAMN02597464, 19 contigs, GenBank Assembly ID: GCA_000517265.1), of *Photorhabdus asymbiotica* subsp. *asymbiotica* ATCC43949 (NCBI reference sequence NC_012962.1), and of the *P. asymbiotica* plasmid pPAU1 (NCBI reference sequence NC_012961.1) were used. LuxR solos in the three above-mentioned *Photorhabdus* species were first identified based on the presence of the C-terminal “HTH LUXR” motif (SMART00421) using SMART 7 software (Simple Modular Architecture Research Tool) (Letunic et al., 2012) and BLAST software (Altschul et al., 1990). Protein domains were identified using SMART 7 software and are identified with a maximal *p*-value of 2e-07 for the “PAS_4”-domain (PFAM08448), of 2e-06 for the “HTH LUXR” motif (SMART00421) and of 1.80e-24 for the “Autoind_bind”-domain (PFAM03472). Furthermore, homologous proteins in *P. asymbiotica* were identified using STRING 9.1 database (Franceschini et al., 2013) based on the LuxR solos of *P. luminescens* (Heermann and Fuchs, 2008).

To elucidate the relationship between the LuxR solos of *Photorhabdus* species, the protein sequences of the 100 LuxR solos of the three *Photorhabdus* species, of LuxR from *Vibrio fischeri*, of TraR from *Agrobacterium tumefaciens*, of SdiA from *Escherichia coli* and of QscR and LasR from *Pseudomonas aeruginosa* were aligned and a phylogenetic tree was generated based on the alignment using CLC Mainworkbench 7 (CLC Bio Qiagen, Hilden, Germany). In the next step, the amino acid residues at the positions of the WYDPWG-motif in the signal-binding domain (SBD) of AHL-sensors were added as metadata layers.

AHL-lactonases and AHL-acylases in the three *Photorhabdus* species were identified using SMART 7 software (Letunic et al., 2012), BLAST software (Altschul et al., 1990) and STRING 9.1 database (Franceschini et al., 2013) based on the presence of known AHL-lactonases and AHL-acylases from *Bacillus cereus, Agrobacterium tumefaciens, Arthrobacter* spec., *Rhodococcus erythropolis, Pseudomonas aeruginosa, Streptomyces* spec., *Dictyoglomus thermophilum*, and *Anabaena* spec. Furthermore, the genomes of the three *Photorhabdus* species were analyzed for the presence of the “Beta-lactamase” motif (PF00144).

## Results and discussion

### *Photorhabdus* species harbor three different types of LuxR solos

**Figure 1:**
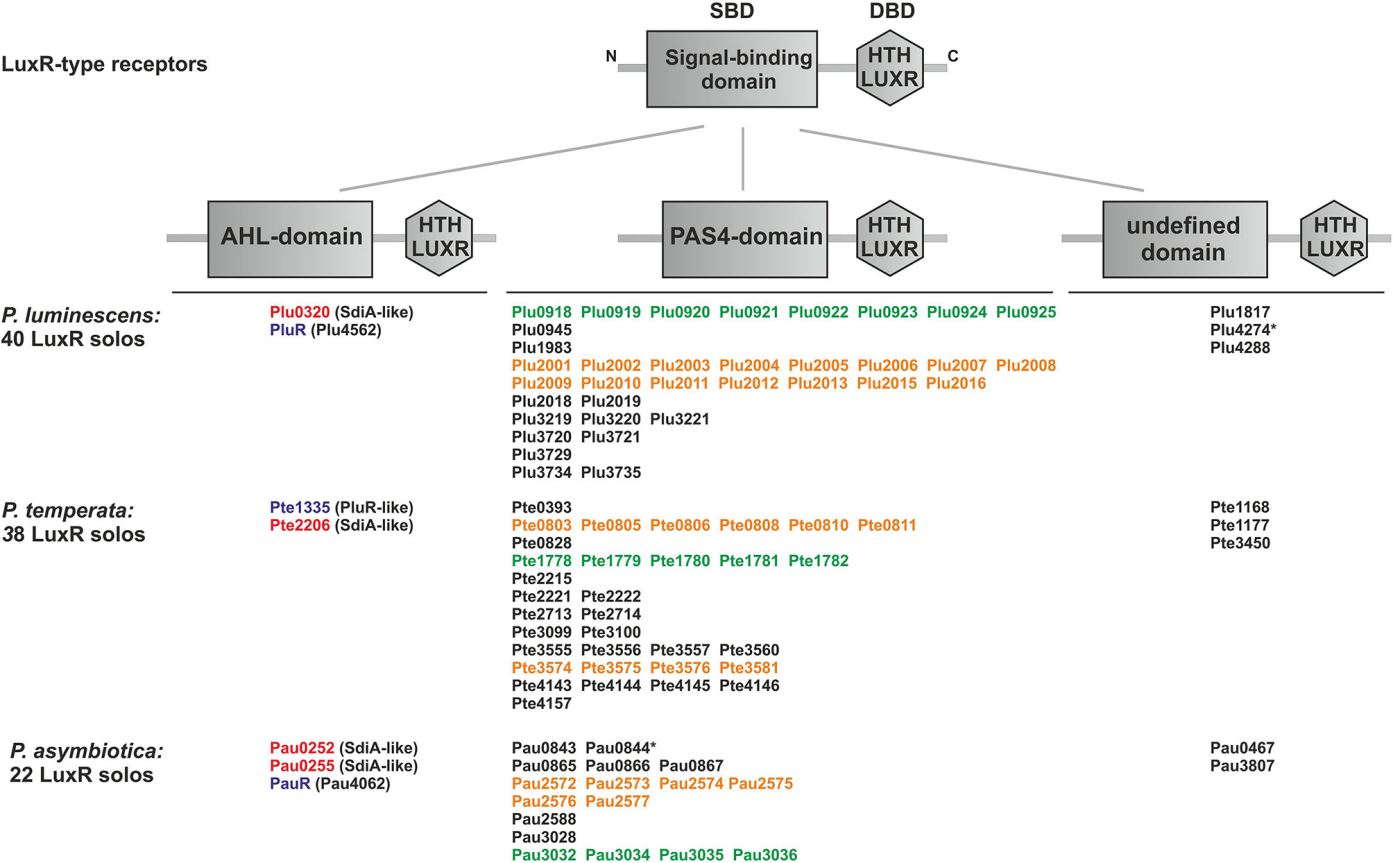
**Presence and domain structure of the LuxR solos in *P. luminescens*, *P. asymbiotica*, and *P. temperata***. LuxR-type receptors share a modular domain structure, with a N-terminal signal-binding domain (SBD) (gray box) and a C-terminal DNA-binding domain (DBD) with the conserved “HTH LUXR” motif (gray hexagon), which is illustrated in the upper part. The N-terminus is marked with an “N” and the C-terminus with a “C.” The LuxR solos of *Photorhabdus* species, *P. luminescens* TT01, *P. temperata* NC19, and *P. asymbiotica* ATCC43949, are grouped into three types based on the different N-terminal domain. The “HTH LUXR” motif (SMART00421) is indicated by a hexagon, the AHL-domain (PFAM03472: “Autoind_bind”-domain) and the PAS4-domain (PFAM08448: “PAS_4”-domain) by boxes. LuxR solos marked with an asterisk additionally have an N-terminal transmembrane domain (not illustrated). Homologous proteins or cluster are marked with the colors red, purple, orange and green. LuxR solos were identified using SMART 7 software (Letunic et al., 2012) and BLAST software (Altschul et al., 1990).

We analyzed the genome of each one representative strain of the three known *Photorhabdus* species *P. luminescens*, *P. temperata*, and *P. asymbiotica* for the presence of genes that encode potential LuxR solos. We found that all three *Photorhabdus* species harbor an exceptionally high number of LuxR solos, with 40, 22, and 38 LuxR solos in *P. luminescens*, *P. asymbiotica*, and *P. temperata*, respectively (Figure 1). A previous study revealed that *P. luminescens* has 39 LuxR solos (Heermann and Fuchs, 2008), but SMART 7 software (Letunic et al., 2012) actually identified Plu4288 as LuxR solo as well, which increased the number of LuxR solos to 40 in this organism. These LuxR solos all contain a C-terminal DNA-binding domain (DBD) with a helix-turn-helix motif, the “HTH LUXR” motif, which is typical for LuxR-type proteins and that was used to identify LuxR solos in *Photorhabdus* species. However, LuxR-type receptors are composed of two functional domains, the C-terminal DBD and the N-terminal SBD that are connected with a short linker (Nasser and Reverchon, 2006). Furthermore, the size of the protein rather than the degree of similarity is crucial whether a protein with such a domain organization meets the criteria to be a LuxR homolog, which is about 250 amino acids in length (Fuqua et al., 1994; Subramoni and Venturi, 2009). All the LuxR homologs identified in the three *Photorhabdus* species meet these criteria, however, diverse domains can make up the SBD, which is the domain that is important for signal-sensing specificity of the receptor. The entire 100 LuxR solos of the three *Photorhabdus* species differ in their N-terminal SBD, for which reason they are grouped into three types: LuxR solos with a PAS4-domain, LuxR solos with an AHL-domain (Autoind_bind) and LuxR solos with yet undefined SBD domains (Figure 1). Presumably, these variable SBD-domains enable the bacteria to sense diverse signals, like exogenous AHLs, exogenous or endogenous non-AHLs, or eukaryotic signals, and thereby influence different cellular processes (Subramoni and Venturi, 2009). The overall number of the LuxR solos might therefore reflect the diversity of invertebrate or vertebrate hosts that *Photorhabdus* species can infect or colonize.

#### LuxR solos containing a PAS4-domain

The majority of the LuxR solos in *Photorhabdus* spec. contain a N-terminal PAS4-domain, which are in sum 80 of the 100 identified LuxR solos. However, the signals sensed by these LuxR solos are yet unidentified. Commonly, PAS-domains are ubiquitous, they appear in archaea, eubacteria and eukarya and are involved in binding of a diverse set of small regulatory molecules either covalently or non-covalently (Hefti et al., 2004). In the fruit fly *Drosophila melanogaster* PAS3 domains have been proved as insect juvenile hormone receptors (Dubrovsky, 2005). The homologous PAS4-domains in *Photorhabdus* are assumed to bind hormone-like molecules and are therefore proposed to be major players in inter-kingdom signaling via the detection of hormone-specific signals from the eukaryotic hosts (Heermann and Fuchs, 2008). As this is most likely, it cannot be excluded that also other bacteria might be sensed via one or more of the PAS4-LuxR receptors. Moreover, the majority of the PAS4-domain-containing LuxR solos in *Photorhabdus* spec. are organized in large gene clusters, which are *plu0915-plu0925* and *plu2001-plu2016* in *P. luminescens*, *pau2572-pau2577*, and *pau3032-pau3036* in *P. asymbiotica* and the operons *pte0803-pte0811*, *pte1178-pte1779*, and *pte3555-pte3581* in *P. temperata*. The function of this genetic clustering as well as the high redundancy of all these PAS4-LuxR solos is unknown to date. Presumably, the high redundancy of PAS4-LuxR solos might be a co-evolutional result by adaptation of the bacteria to the variety of insect hosts they can infect. This idea is underlined by the finding that several LuxR solos from plant-associated bacteria are known to respond to plant signaling molecules and are therefore assumed to have undergone co-evolution with the related host plant (Covaceuszach et al., 2013). Putative signals sensed by the PAS4-LuxR solos might be diffusible substances like hormones. The phytopathogens belonging to the genus *Xanthomonas* use a fatty acid signal belonging to the Diffusible Signal Factor (DSF) family for cell-cell signaling to control the virulence of *Xanthomonas campestris* pv. *campestris* (Xcc) to plants. Recently, a second sensor for DSF was identified, which is a complex sensor kinase having a N-terminal PAS4-domain essential for sensing of DSF (An et al., 2014). This supports the idea that the PAS4-LuxR solos of *Photorhabdus* spec. are adapted to distinct signals from the invertebrate hosts (insects and nematodes) or in case of *P. asymbiotica* additionally to vertebrate hosts, especially humans, to expand or fine-tune their regulatory network.

#### LuxR solos containing an AHL-domain

Besides the high number of PAS4-LuxR solos, all three *Photorhabdus* species contain at least one LuxR solo that is homologous to the LuxR solo SdiA. *P. luminescens* and *P. temperata* each have one SdiA homolog, Plu0320 and Pte2206, respectively, whereas the human pathogenic *P. asymbiotica* has two SdiA homologs, Pau0252 and Pau0255. The LuxR solos SdiA of *Escherichia coli* and *Salmonella enterica* are known to detect exogenously produced AHLs and therefore these organisms are able to sense mixed microbial communities (Wang et al., 1991; Michael et al., 2001). All four SdiA homologs in *Photorhabdus* spec. have a N-terminal “Autoind_bind” domain (PFAM03472) (Figure 1), which is typical for AHL-sensors and responsible for AHL-sensing. Similar to SdiA, the four LuxR solos Plu0320, Pau0252, Pau0255, and Pte2206, respectively, can be assumed to detect AHLs as well. Since no *luxI* gene is present in any of the *Photorhabdus* genomes, it is most likely that these LuxR solos can respond to exogenously produced AHLs, produced by other bacteria, probably of the insect gut, which are then sensed by *Photorhabdus* spec. during the infection process. Moreover, AHLs have never been detected using different analytical chemistry methods (HPLC/MS, GC/MS) in any *Photorhabdus* strain, despite the analysis of >100 strains (Darko Kresovic and Helge B. Bode, data not shown). As the human pathogen *P. asymbiotica* has two putative AHL-sensors (Figure 1), it is most likely that AHL-sensing plays an even more important role for vertebrate than invertebrate host infection. Interestingly, also the three homologous LuxR solos PluR (Plu4562) of *P. luminescens*, PauR (Pau4062) of *P. asymbiotica*, and PluR (Pte1335) of *P. temperata* contain a predicted “Autoind_bind” domain like the AHL sensors. However, the LuxR solo PluR senses α-pyrones named photopyrones (PPYs) instead of AHLs (Brachmann et al., 2013), which shows that the presence of an “Autoind_bind” domain is not automatically going along with AHL-sensing. PluR was the first example of a LuxR solo that senses an endogenous signal and is therefore part of a bacterial cell-cell communication system in *P. luminescens* regulating expression of the *pcfABCDEF* operon leading to cell clumping (Brachmann et al., 2013). *P. luminescens* produces eight different PPYs, out of which three (PPYA, PPYB, PPYD) are mainly present in the culture fluid. Among these, PluR senses PPYD with the highest sensitivity in a concentration as low as 3.5 nM. Furthermore, the ketosynthase-like protein PpyS was identified as the PPY synthase and therefore as producer of the signaling molecules. All proteins are also present with high homology in *P. temperata*, which indicates a similar strategy for cell-cell communication in this genus (Brachmann et al., 2013). The insect and human pathogen *P. asymbiotica* harbors a *pcf* regulon that is highly homologous to that of *P. luminescens*. It consists of the *pcfABCDEF* (*pau4068-pau4063*) operon and an adjacent gene encoding a LuxR solo that we named PauR (Pau4062). However, *P. asymbiotica* contains neither a *ppyS* homolog nor a *luxI*-like synthase and does not produce any PPYs or AHLs. Therefore, it is assumed that *P. asymbiotica* uses yet unidentified signaling molecules for cell-cell communication, which are sensed by PauR. As we will focus on below, this is rather due to the presence of a conserved amino acid motif within the SBD, which is intact in the SdiA homologs, but different in both PluR proteins and PauR. When PluR was originally detected it met the criteria to be defined as LuxR solo. Since PluR has a cognate synthase, but which is different from LuxI, it is thus part of an entire cell-cell communication system. Therefore, it is disputable whether the designation LuxR solo is actually appropriate at least for PluR.

#### LuxR solos containing a non-defined signal-binding domain

Besides LuxR solos with defined SBDs, all three *Photorhabdus* species harbor LuxR solos with yet unidentified SBDs, namely Plu1817, Plu4274, Plu4288, Pau0467, Pau3804, Pte1168, Pte1177, and Pte3450. In contrast to many other LuxR solos, which are often hydrophobic and membrane associated (Welch et al., 2000), Pau0844 and Plu4274 are predicted to contain a N-terminal transmembrane domain (TM). Although the exact mechanism of DNA-binding by a membrane-integrated protein is not yet fully understood, other examples of those membrane-integrated DNA-binding receptors are known, e.g., the pH-sensor CadC of *Escherichia coli* (Haneburger et al., 2011). The presence of an intact TM within the SBD could give indication for sensing an extremely hydrophobic and non-water-soluble signaling molecule. The LuxR solo Pau0844 is the only LuxR solo that harbors a TM in combination with a PAS4-domain, which might therefore sense an extremely hydrophobic eukaryotic signal. Overall, the LuxR solos with yet undefined SBDs might expand the range of signals that are sensed and therefore the communication network. However, their roles in cell-cell communication and/or inter-kingdom signaling as well as the related signaling molecules are unknown to date.

### Conserved amino acid motifs in the signal-binding domain of LuxR solos

**Figure 2:**
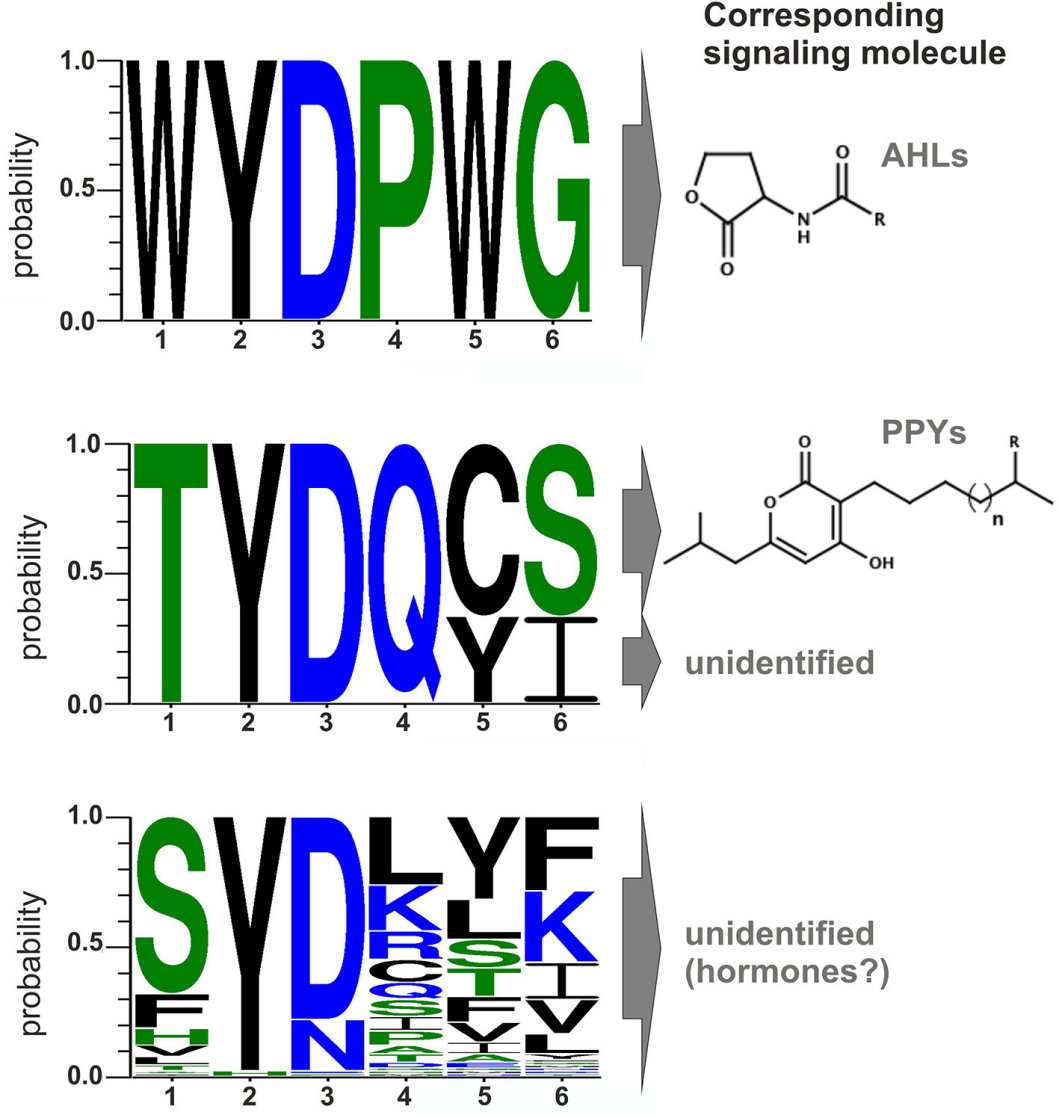
**Conserved amino acid motifs of LuxR-type proteins with different domains and their corresponding signaling molecules. Upper part**: Motif of the six conserved amino acid positions in typical AHL-sensors. Protein sequences of LuxR from *Vibrio fischeri*, TraR from *Agrobacterium tumefaciens*, SdiA from *Escherichia coli*, QscR and LasR from *Pseudomonas aeruginosa* were used to generate the alignment. **Middle part**: Motif of the six conserved amino acids positions of PluR (Plu4562) from *P. luminescens*, PluR (Pte1335) from *P. temperata* and PauR (Pau4062) from *P. asymbiotica*. PluR from *P. luminescens* and from *P. temperata* are sensing photopyrones as signaling molecule, however this is yet unidentified for PauR. **Lower part:** Motif of the six conserved amino acid positions of the overall 80 PAS4-LuxR solos in all three *Photorhabdus* species, whereas the corresponding signal molecules are yet unknown but possible are eukaryotic hormones. All alignments were generated with CLC Mainworkbench 7 software (CLC Bio Qiagen, Hilden, Germany). The sequence logo was made with WebLogo3 (Crooks et al., 2004).

Comparison of all LuxR solos of *Photorhabdus* spec. indicates that signal-specificity relies on the SBD, given that the major differences are located in the SBD and the similarities in the DBD. In general, proteins of the LuxR family are less conserved in their amino acid sequence, which only makes up between 18 and 25% identity (Subramoni and Venturi, 2009). However, LuxR-type proteins sensing AHLs harbor six highly conserved amino acid in the N-terminal SBD that are important for signal-binding, signal molecule specificity and shaping the signal-binding pocket. These six conserved amino acids are W57, Y61, D70, P71, W85, and G113 (with respect to TraR), which are either hydrophobic or aromatic and displaying a conserved motif for AHL-sensors (Patankar and González, 2009). Bioinformatics analyses of the amino acids of the *Photorhabdus*-specific LuxR solos at these positions reveals that the conserved WYDPWG-motif of AHL-sensors is only completely present in the SdiA homologs Plu0320 of *P. luminescens*, Pau0252 and Pau0255 of *P. asymbiotica*, and Pte2206 of *P. temperata*, supporting the idea that these LuxR solos sense exogenous AHLs. All other LuxR solos in *Photorhabdus* spec. have at least two substitutions at different positions in the WYDPWG-motif. Remarkably, amino acid Y61 (with respect to TraR) is present in 97% of the LuxR solos of the three *Photorhabdus* species. This amino acid is known to be involved in binding of the acyl chain of the signaling molecule via hydrophobic interactions, e.g., in TraR (Churchill and Chen, 2011) or LuxR (Nasser and Reverchon, 2006). Therefore, there seems to be a common mechanism to arrange distinct signaling molecules in the signal-binding pocket, independent from the exact chemical nature of the signaling molecule. The position D70 (with respect to TraR) is known to be important for binding the amide group of the cognate AHL within AHL-binding LuxR receptors (Churchill and Chen, 2011). This position is also conserved among 80% of the LuxR solos in the three *Photorhabdus* species, whereas the remaining LuxR solos (19%), mainly from *P. asymbiotica*, have the substitution D70N at this position, which might be important to sense eukaryotic signals during vertebrate infection (Figure 2). Although not sensing AHLs (Brachmann et al., 2013), the three LuxR solos, PauR and both PluR homologs of *P. asymbiotica*, *P. luminescens*, and *P. temperata*, respectively, have a predicted “Autoind_bind” domain. However, only the two conserved amino acids Y61 and D70 (with respect to TraR) are present in these LuxR solos. Moreover, both PluR proteins share a TYDQCS-motif, which is therefore probably specific for PPY-sensing (Figure 2). Individual replacement of Y66 and D75 with alanine within PluR prevented PPY-binding, revealing that these amino acids are important for PPY-binding and/or specificity (Brachmann et al., 2013). In contrast, PauR has a slightly different SBD-motif, which is TYDQYI, indicating that this LuxR solo, although highly homologous to both PluR proteins, senses a signal that is different from PPYs as well as AHLs. Overall, variations in the WYDPWG-motif that is typical for AHL-sensing probably expand the diversity of signals that can be sensed by LuxR-type proteins having an “Autoind_bind” domain. Additionally, the 80 LuxR solos of *Photorhabdus* spec. with a PAS4-domain share a more variable amino acid motif in the SBD, reflecting a higher variety of signals they might sense (Figure 2). However, the precise signals that are sensed by PAS4-LuxR solos are yet unknown. The huge diversity of the SBD-motifs of PAS4-LuxR solos in *Photorhabdus* and the variations in the conserved amino acid motifs probably gives the bacteria the capacity to respond to a broad range of signals that occur in the different environments or hosts.

**Figure 3:**
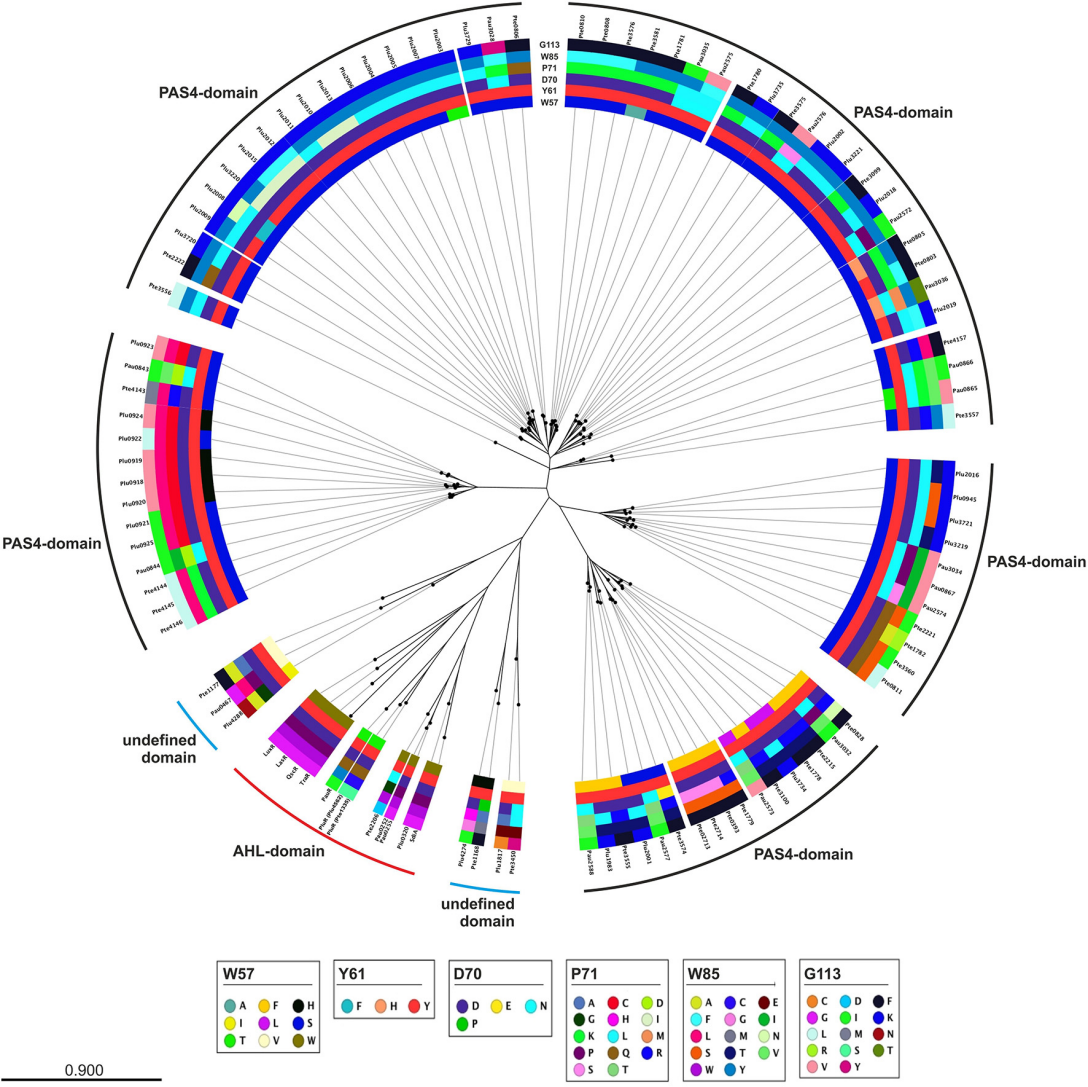
**Phylogenetic tree of the LuxR solos present in *P. luminescens, P. temperata*, and *P. asymbiotica.*** Protein sequences of the overall LuxR solos of the three *Photorhabdus* species, of LuxR from *Vibrio fischeri*, of TraR from *Agrobacterium tumefaciens*, of SdiA from *Escherichia coli* as well as of QscR and LasR from *Pseudomonas aeruginosa* were aligned and a phylogenetic tree was generated. Based on this alignment the different amino acid motifs at the six conserved positions were identified by deviation from the WYDPWG-motif in the SBD of AHL-sensors. A special focus on the amino acids at positions of the WYDPWG-motif is shown from the inner to the outer circle. The amino acid W57, with respect to TraR, is marked in brown, Y61 in red, D70 in purple, P71 in dark red, W85 in pink and G113 in light pink, however substitutions within this positions are marked in different colors. LuxR-type proteins with an AHL-binding domain are marked with a red dash, LuxR-type proteins with a PAS4-domain are marked with a black dash and LuxR-type proteins with a N-terminal yet undefined domain are marked with a blue dash. Alignment and radial phylogenetic tree was generated with the CLC Mainworkbench 7 (CLC Bio Qiagen, Hilden, Germany). The scale bar indicates the length of the branches.

A closer look at the protein sequence and phylogeny of the *Photorhabdus*-specific LuxR solos reveals that related LuxR solos, independent of their host organism, share the similar amino acid motif in the SBD (Figure 3). More precisely, LuxR solos encoded by one operon tend to have a similar amino acid motif at the six conserved positions of the SBD. For example the *plu0919-plu0925* operon and the homologous *pte4143-pte4146* operon encoding the respective PAS4-LuxR solos both show replacement of amino acid W85 (with respect to TraR) to L and of G113 (with respect to TraR) either to V or L in the WYDPWG-motif. This in total applies for 14 of 80 LuxR solos that have a PAS4-domain. However, over 30 LuxR solos show a substitution of W85 to Y containing a PAS4-domain. These homologous changes may originate via gene duplications within one species and might give these LuxR solos the ability to sense similar signals. On the other hand, closely related LuxR solos also incline to the same kind of substitution of the amino acid motif in the SBD (Figure 3). The homologous PluR LuxR solos, Plu4562 from *P. luminescens* and Pte1335 from *P. temperata*, share the identical substitutions of four conserved amino acids, forming a TYDQCS-motif allowing both to sense PPYs. The closely related LuxR solo PauR from *P. asymbiotica* harbors the slightly different TYDQYI-motif at the similar positions, suggesting binding of a distinct signaling molecule. This could be due to the fact that cell-cell-communication via PPY might be inappropriate when infecting vertebrates. Comparably, the LuxR solos of the novel subfamily of plant-associated bacteria have substitutions in the WYDPWG-motif, which are W57M and Y61W (with respect to TraR) that might allow the binding of plant signal molecules rather than AHLs (Patankar and González, 2009; González and Venturi, 2013). The *P. luminescens* specific PAS4-LuxR solos Plu2018, Plu3221, Plu3720, Plu3729, and Plu3735 are closely related to each other and all have an identical amino acid motif within the SBD, which is SYDLYK. This shows that phylogeny of the LuxR solos correlates with the protein sequence as well as the six conserved amino acid positions within the SBD.

Furthermore, variations of the amino acids at the conserved positions to motifs different from WYDPWG offers the possibility to sense different signals or altering the affinity toward a specific signal, as the latter is true for EsaR from *Pantoea stewartii* (Shong et al., 2013). However, LuxR solos with similar amino acid motifs in the SBD of different species might in turn lead to the binding of the same signaling molecules.

### Quorum quenching via AHL-lactonases and AHL-acylases in *Photorhabdus* species

**Figure 4:**
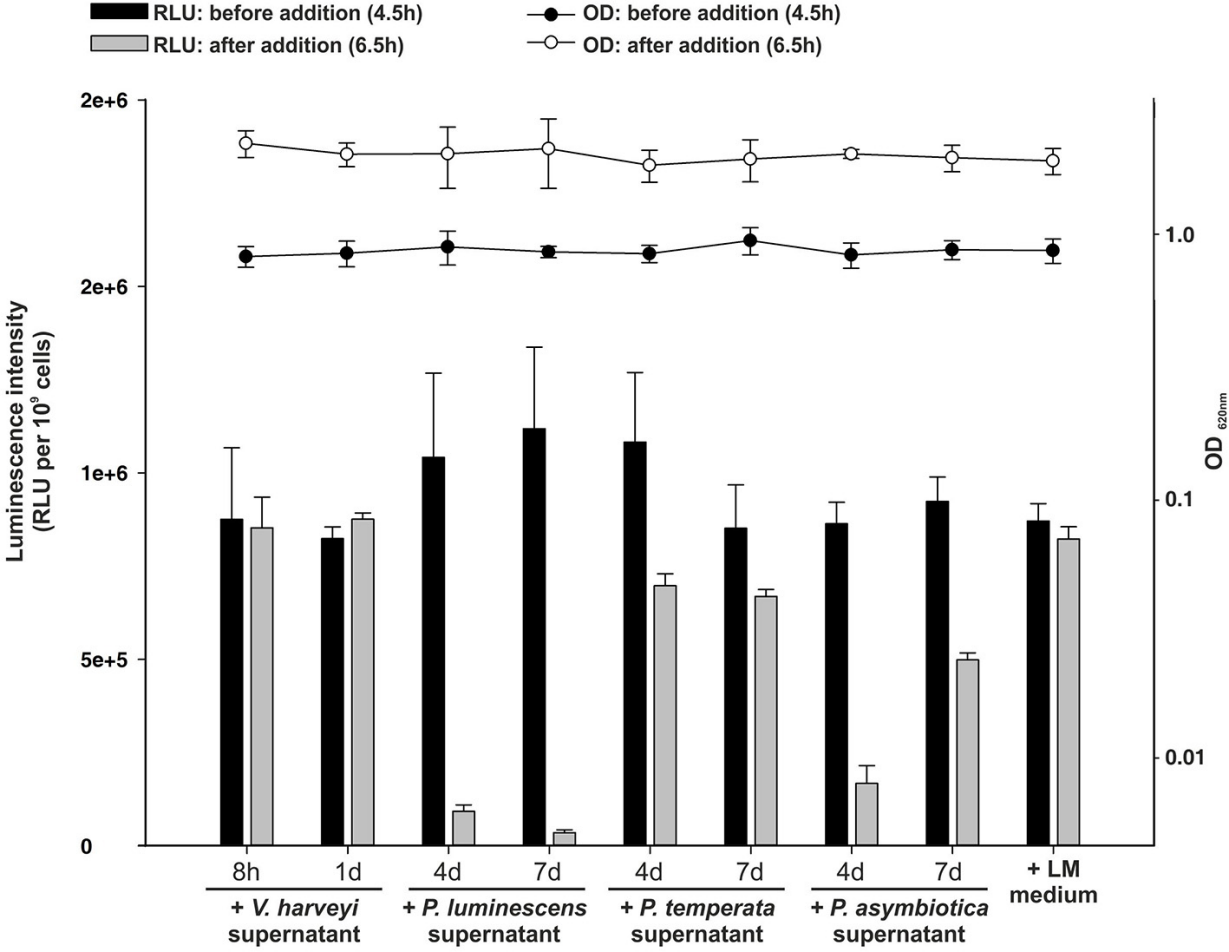
**Quorum quenching by *Photorhabdus* species culture fluids**. Bioluminescence of *Vibrio harveyi* wild-type BB120 was used as read-out to analyze degradative activity of *Photorhabdus* spec. supernatants. The supernatants of *P. luminescens*, *P. temperata*, and *P. asymbiotica* were harvested after 4 and 7 days of growth and added to the BB120 reporter strain in the mid-exponential growth phase, when bioluminescence occurs. As controls *V. harveyi* supernatant and LM medium, harvested after 8 h and 1 day of growth, was added. Luminescence (RLU) was measured before (black bars) and after (gray bars) the addition of the *Photorhabdus* spec. supernatant and optical density (OD) was measured before (black circles) and after (white circles) the addition of the *Photorhabdus* spec. supernatant. Error bars represent standard deviation of at least three independently performed experiments. RLU, relative light units.

As identified above, each *Photorhabdus* species harbor a SdiA-homolog, which are the LuxR solos Plu0320, Pte2206, Pau0252, and Pau0255 of *P. luminescens*, *P. temperata*, *and P. asymbiotica*, respectively, containing an AHL-binding domain. Although the functions of these LuxR solos are yet unknown, it might be the sensing of external AHLs. One reason for sensing external AHLs could be to interfere with LuxR quorum sensing systems of their environment by degradation of the QS signaling molecule of neighboring bacteria. This process is designated as quorum quenching (QQ), interferes with the communication of a bacterial population, and can be used to influence their activity, e.g., virulence. For this purpose, many bacteria produce lactonases and acylases, which hydrolyze the lactone ring and cleave the amide bond, respectively, of AHLs. These enzymes are found in both Gram-negative AHL producers and non-AHL producers as well as in Gram-positive bacteria (Williams, 2007). The virulence of the plant pathogens *Pseudomonas solanacearum*, diverse *Erwinia* species and the human pathogen *Pseudomonas aeruginosa* is influenced via QQ enzymes (Dong et al., 2000). We analyzed the genome of all three *Photorhabdus* species for the presence of genes that encode putative AHL-lactonases and AHL-acylases. We identified several homologs of known AHL-lactonase and AHL-acylases in all three *Photorhabdus* species (Table 1). Particularly, homologs for the AHL-lactonase AiiA from *Bacillus* spec., which inactivates the QS signal and decreases the virulence of *Erwinia carotovora* (Park et al., 2008), are found in all three *Photorhabdus* species, which are Plu1749, Pau1378, and Pte3377. In addition, AiiA showed a strong enzymatic activity toward distinct AHLs, which vary in length and the substitution at the C3 position of the acyl chain (Wang et al., 2004). Park et al. (2003) demonstrated that the AHL-lactonase AhlD of *Arthrobacter* spp. blocks the communication of other bacteria and yet *P. luminescens* could use the same strategy via the homolog Plu2238. The AHL-lactonase AttM of *A. tumefaciens*, encoded on the At plasmid, is important for the attachment to plant cells (Liu et al., 2007) and a homolog is found in each *Photorhabdus* species, which are Plu2238, Pau0481, and Pte1161. However, the plasmid pPAU1 from *P. asymbiotica* harbors no known AHL-lactonase or AHL-acylase or homologs revealing that QQ by *Photorhabdus* is not especially important for vertebrate infection, or that the similar QQ enzymes for vertebrate as well as for invertebrate infection are used. However, we not only detected the presence of these putative AHL-lactonases and AHL-acylases in *Photorhabdus* species. Moreover, we analyzed the potential QQ ability of *Photorhabdus* species culture fluids. Therefore, the culture fluids of all three *Photorhabdus* spec. at different growth phases were examined using the marine bacterium *Vibrio harveyi* and its regulation of bioluminescence via accumulation of the AHLs as reporter. Thereby, all three culture fluids emerged to have high QQ activity, putatively by degradation of the AHL HAI-1 of *V. harveyi* (Figure 4). Especially, the culture supernatant of *P. luminescens* had the highest AHL-degrading activity against HAI-1 of *V. harveyi* compared to those of *P. temperata* as well as *P. asymbiotica* (Figure 4). However, the number of putative AHL-lactonases and AHL-acylases homologs predicted in all three *Photorhabdus* species is similar (Table 1). These differences in QQ could therefore only be explained with the theory that the respective genes might be differently regulated in the three *Photorhabdus* species and that therefore not all enzymes are fully produced or activated under the *in vitro* experimental conditions used. Anyway, our findings indicate that the three *Photorhabdus* species can interfere with the AHL-based quorum sensing systems of other bacteria. This could be an important step in the infection process, probably to shut down virulence of opportunistic pathogens or food competitors that are present in the host gut or in the soil to prevent them from pitching into their prey or to stop them from antibiotic production. The expression of the different AHL-lactonases and/or AHL-acylases encoding genes could be activated via the SdiA-homologous LuxR solos Plu0320, Pte2206, Pau0252, and Pau0255 of *P. luminescens*, *P. temperata*, *and P. asymbiotica*, respectively, after sensing the external AHLs. However, whether the AHL-lactonases and/or AHL-acylases encoding genes are under control of the SdiA-homologous LuxR solos in *Photorhabdus* spec. is indeed possible, but has to be investigated.

**Table 1:** **Homologous AHL-lactonases and AHL-acylases in *Photorhabdus* spec**.

**Strain**	**Protein**	**Enzymatic activity**	***P. luminescens* TT01**	***P. asymbiotica* ATCC43949**	***P. temperata* NC19**
*B. cereus*	AiiA	AHL-lactonase	Plu1749	Pau1378	Pte3377
*A. tumefaciens*	AiiB	AHL-lactonase	–	AttM/AiiB (Pau0481)	–
*A. tumefaciens*	AttM	AHL-lactonase	Plu2238	AttM/AiiB (Pau0481)	Pte1161
*Arthrobacter* spec.	AhlD	AHL-lactonase	Plu2238	–	–
*P. aeruginosa*	PvdQ	AHL-acylase	Plu3527	–	–
*Streptomyces* spec.	AhlM	AHL-acylase	Plu3527	–	–
*D. thermophilum*	AhlA	AHL-acylase	Php (Plu1997)	Php (Pau2581)	Pte3570
*Anabaena* spec.	AiiC	AHL-acylase	Plu3527	–	–

*The AHL-lactonases AiiA from *Bacillus cereus* (Wang et al., 2004), AiiB from *Agrobacterium tumefaciens* (Liu et al., 2007), AttM from* Agrobacterium tumefaciens *(Zhang et al., 2002) and AhlD from* Arthrobacter spec. *(Park et al., 2003) were used in this study to identify homologs in the three* Photorhabdus *species. AHL-lactonases have a “Lactamase_B” domain (PF00144) belonging to the metallo-beta-lactamase superfamiliy. Furthermore, Zn^2+^-dependent hydrolases exist, e.g., AiiB, AhlD, AttM, QsdA, Plu2238, Pau1378, Pte1161, and Pte3377. The acylases PvdQ from* Pseudomonas aeruginosa *(Huang et al., 2003), AhlM from* Streptomyces spec. *(Park et al., 2005), AhlA from* Dictyoglomus thermophilum *(Coil et al., 2014), and AiiC from* Anabaena spec *(Romero et al., 2008). Analyses are based on STRING 9.1 database and NCBI Blast (Altschul et al., 1990) were used in this study to identify homologs in the three* Photorhabdus *species. The AHL-acylases AhlM, AhlA, and AiiC belong to the Ntn-hydrolase superfamily.*

Overall, the defense of the dead insect larvae, which is a rich food source, is crucial for the survival of *Photorhabdus* as well as the nematode partner. Thus, disturbing of the ambient mixed microbial community and its communication might be an important step for a successful infection process and reproduction.

## Conclusions

**Figure 5:**
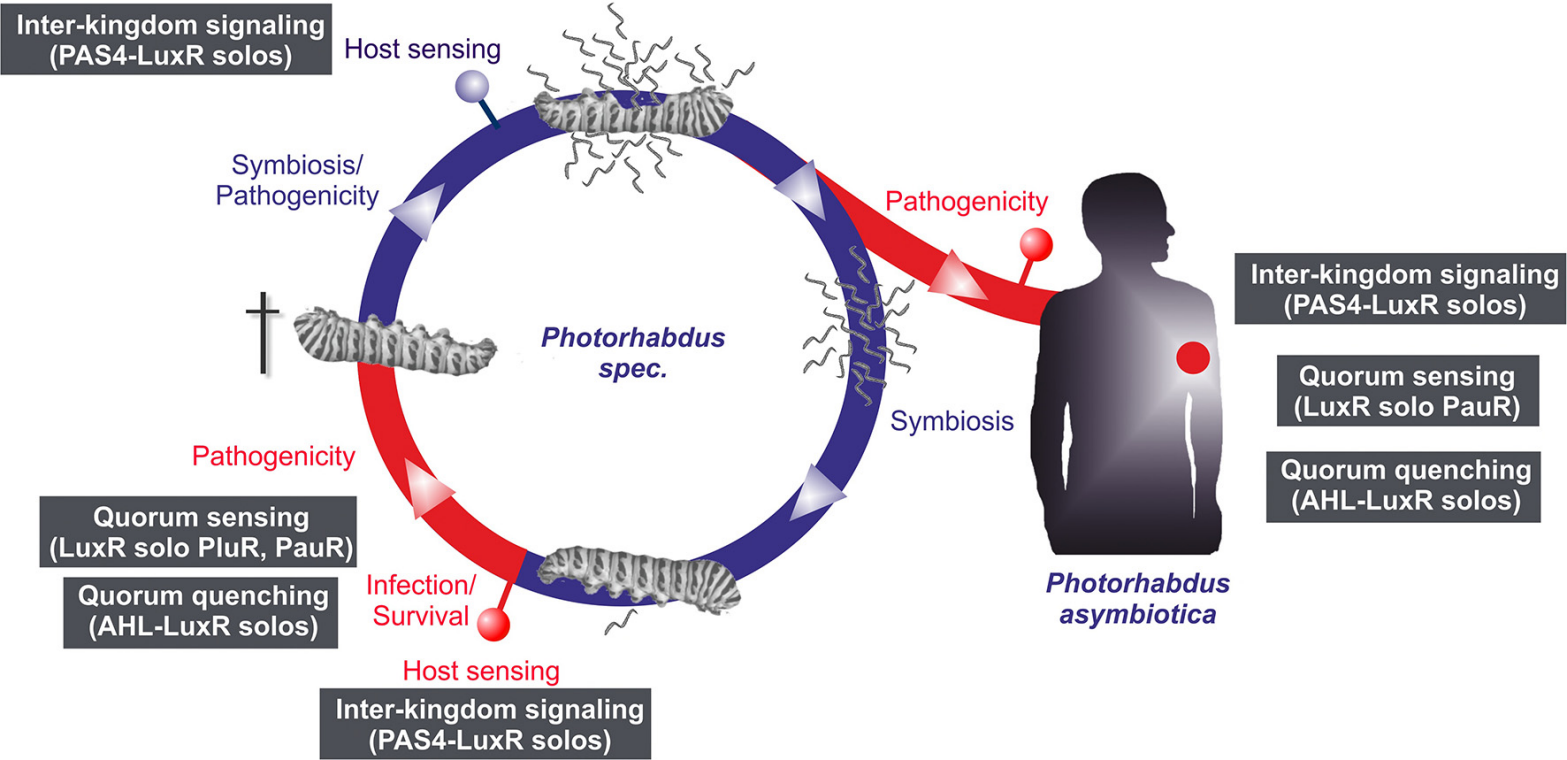
**Life and infection cycle of *Photorhabdus* spec. and involvement of the different LuxR solos**. Life cycle of *P. luminescens*, *P. temperata*, and *P. asymbiotica* (left panel). The bacteria colonize the upper gut of heterorhabditid nematodes that invade insect larvae. After release into the insect's hemolymph the bacteria produce several toxins that rapidly kill the prey. After death of the insect host, the bacteria degrade the cadaver, and additionally support nematode development. When the cadaver is depleted from nutrients, bacteria and nematode re-associate and leave the carcass in search for a new victim. The pathogenic part of the cycle is drawn in red, the symbiotic part in blue. *P. asymbiotica* can additionally infect humans by inducing systemic and soft tissue infections (right panel). Putative involvement of the different LuxR solos at steps of the infection process or host sensing is indicated by the gray boxes (see text for details).

LuxR solos emerge to be more and more important players in cell-cell communication or inter-kingdom-signaling as they offer possibilities using alternative communication molecules to AHLs. In all three *Photorhabdus* species an extraordinary high number of LuxR solos were identified, making them to optimal model organisms to study the function of LuxR solos in bacteria. Thereby, regulation via LuxR solos can be proposed to be important at different steps of the *Photorhabdus* life cycle and infection process (Figure 5). However, the majority of the LuxR solos in all three *Photorhabdus* species contain a PAS4-domain, which lends support for the theory that host sensing is highly important at different steps in the *Photorhabdus* life cycle. In contrast, sensing other bacteria by detecting their AHLs as well as quorum sensing seems to be only important for the pathogenic steps of the life cycle rather than for the symbiosis parts. We have seen that the LuxR solos contain different SBDs, which include diverse amino acid motifs at conserved positions. The diversity of these motifs gives rise to the speculation that signal-binding of all these LuxR solos goes far beyond AHL-signaling, as we have recently demonstrated for PluR for the first time (Brachmann et al., 2013). One can only guess the variety of signals perceived by all these LuxR solos and their function in cell-cell communication and inter-kingdom-signaling. It will be the goal of the near future research to unravel the various signaling molecules and to correlate them to the specific LuxR solos or amino acid motifs in the SBD of these proteins. The presence of all those different types of LuxR solos gains first insight into the complexity of the communication network between bacteria among each other as well as with their hosts. As most of the LuxR solos that have been investigated so far are involved in regulation of insect or plant pathogenicity, the homologous receptors or the related signaling molecules might be promising specific drug targets in human pathogens as well.

### Conflict of interest statement

The authors declare that the research was conducted in the absence of any commercial or financial relationships that could be construed as a potential conflict of interest.
